# Prognostic impact of fibrosclerotic changes in non-papillary, non-anaplastic, follicular cell-derived thyroid carcinomas

**DOI:** 10.1007/s00428-025-04028-2

**Published:** 2025-01-23

**Authors:** Giulia Orlando, Giulia Capella, Giulia Vocino Trucco, Elena Vissio, Jasna Metovic, Francesca Maletta, Marco Volante, Mauro Papotti

**Affiliations:** 1 Department of Oncology, University of Turin, Orbassano, Turin, Italy; 2 Department of Medical Sciences, University of Turin, Turin, Italy; 3 Pathology Unit, Savigliano Hospital, Savigliano, Cuneo, Italy; 4 Pathology Unit, Città Della Salute E Della Scienza Hospital, Turin, Italy

**Keywords:** Thyroid carcinoma, Follicular, Poorly differentiated, Fibrosis, Prognosis

## Abstract

In non-papillary follicular cell-derived thyroid carcinomas, prognostic factors are scarce. Intratumoral fibrosis was identified as an adverse factor in papillary and medullary carcinomas, but it has not been investigated in other subtypes. We aimed at exploring the presence of intratumoral fibrosclerosis in a cohort of 132 non-papillary follicular cell-derived thyroid carcinomas (53 follicular and 31 oncocytic carcinomas, including 10 high grade differentiated thyroid carcinomas and 48 poorly differentiated carcinomas) and correlating its presence and extent with clinical and pathological features and survival. For each case, all available hematoxylin and eosin slides were reviewed, and the presence of fibrosclerosis was assessed as the percentage of tumor area and semi-quantitatively scored as absent, mild (≤ 10%) or extensive (> 10%). In addition, digital image analysis was applied in 65 cases. Scoring of intratumoral fibrosis showed a strong agreement between two observers and between observers and digital image quantification. The presence and extent of intratumoral fibrosis were significantly associated with poorly differentiated carcinoma histology, large tumor size, extent of vascular invasion, presence of necrosis, high mitotic index, positive nodal status, and aggressive clinical outcome, and with a shorter disease-free and disease-specific survival, the former also in follicular and oncocytic carcinomas analyzed separately. These data support the potential use of fibrosis in the clinical practice since it is both easily assessable and significantly associated with the presence of parameters of aggressiveness. In addition, fibrosis is correlated with decreased survival rate independently from the tumor histotypes, suggesting its potential role as novel prognostic factor in non-papillary follicular cell-derived thyroid carcinomas.

**Supplementary Information:**

The online version contains supplementary material available at 10.1007/s00428-025-04028-2.

## Introduction

At variance with papillary thyroid carcinoma, both follicular carcinoma (FC) and oncocytic carcinoma (OC) of the thyroid are poor of prognostic factors that may assist in their risk stratification and that may help to define the most appropriate clinical management. In a recent meta-analysis, age > 45 years, male sex, tumor size > 4 cm, multifocality, extrathyroidal extension, widely invasive subtype, and the presence of cervical lymph node metastasis and/or distant metastases were identified as the most relevant factors that increase the risk of death of patients with FC [1]. Age at diagnosis, pT3/pT4 stage, presence of lymph node metastases, widely invasive tumors, and extra-thyroidal extension were also confirmed as parameters increasing the risk of radio-iodine refractory disease [2]. Moreover, the majority of cases are represented by encapsulated angioinvasive carcinomas, with a prognostic sub-stratification based exclusively on the number of blood vessels involved by the tumor. Although generally a cut off of 4 blood vessels has been used for prognostic stratification [3, 4], recently, a cut off of 2 has been proposed by Japanese authors as an independent negative prognostic factor for disease-free survival, together with age ≥ 55 years, histological subtype and tumor size > 4 cm [5].

The presence of widely invasive subtype and extensive vascular invasion is also relevant negative prognostic factors in OC [6–8], together with age > 55 years, male sex, and higher tumor stage [9].

Within the group of high-grade follicular cell-derived thyroid carcinoma (HG-FCDTC), poorly differentiated carcinoma (PDC) subtype possesses a specific tumor architecture and clinical pathological characteristics. In this subtype, the extent of invasion has been also shown to affect disease-free survival [10], whereas the lack of encapsulation has been associated with increased risk of disease-related death [11], together with age > 45 years and size > 4 cm [12]. Moreover, oncocytic features, even focal, are associated with decreased survival rates and increased radio-iodine refractory disease [13].

Apart for pure pathological characteristics, the presence of molecular alterations has a low impact in the prognostic stratification of FC, OC, and PDC. In FC, mutation burden but not *RAS* mutations was shown to be associated with prognosis [14]. The presence of *TERT* promoter mutations, only, proved to be a negative prognostic indicator in FC [15], but its clinical value is limited by the very low prevalence of such alterations [16], thus limiting its sensitivity as a clinically applicable biomarker. High molecular-risk signature using the ThyroSeq v3 panel was associated with the presence of distant metastasis and poor overall survival in PDC [17].

Among pathological parameters potentially bearing a prognostic impact, intratumoral fibrosis has been investigated in thyroid cancer, but most data are restricted to papillary carcinoma (PC) [18]. Xu and co-workers showed that in a high percentage of PC having low-risk histology and presence of distant metastases, extensive intra-tumoral fibrosis occurred thus suggesting that fibrous bands could be predictive of a more aggressive behavior in this histotype [19]. Similarly, Takeda and co-workers demonstrated that PC cases with nodular fibrosis had a statistically significant increment of the risk of developing lymph node metastasis, multifocal lesions, and extra-thyroidal invasion as compared to non-fibrotic PC [20], possibly as the result of the activation of hypoxia-inducible factor 1α pathway [21].

Few data are available on the significance of intratumoral fibrosis in the other non-papillary thyroid carcinoma subtypes. This is more probably related to the low prevalence of non-papillary carcinoma histotypes that hampers the analysis of large series. This study by Xu and coworkers mentioned above [19] included one case of FC and 2 cases of OC but it is not clear whether these cases had or not intratumoral fibrosis. Rivera and coworkers [22] described extensive intratumoral fibrosis in minimally invasive FC with distant metastases. The authors suggested that these findings are in line with the data of Collini [23] and Lang [24], although these publications did not address specifically the presence of intratumoral fibrosclerotic changes. Moreover, a study by Cracolici and co-workers described that FC with distant metastases have a high prevalence of intratumoral fibrosis (6/18 cases overall tested) and that the presence of intratumoral fibrosis is associated with a clinical presentation of distant metastases prior to the identification of the thyroid tumor, as compared to cases presenting with a thyroid mass with metachronous distant dissemination [25]. However, a detailed description of how fibrosis was estimated in this study is not provided. Taking all these data together, it is conceivable that intratumoral fibrosis might be a clinically relevant pathological parameter helping to define aggressiveness also in non-papillary carcinoma histotypes. However, research needs in this files include data from large and well characterized series, a clear definition of criteria to define the presence and extent of intratumoral fibrosis and the exploration of easily accessible digital image tools to mitigate interobserver variability.

Based on the above, the aim of our work was to explore the presence of intratumoral fibrosclerotic changes in a large series of non-papillary, non-anaplastic, follicular cell-derived thyroid carcinomas, to test interobserver variability and the usefulness of digital image analysis, and to correlate the presence and extent of intratumoral fibrosis with clinical and pathological characteristics.

## Material and methods

### Case series

From the pathology files of the “Città della Salute e della Scienza” academic hospital (Turin, Italy), a non-consecutive series of 132 surgical specimens of non-papillary, non-anaplastic, follicular cell-derived thyroid cancers was retrospectively selected from years 2000 to 2017. The cases consisted of 53 follicular carcinomas (FC), 31 oncocytic carcinomas (OC), and 48 poorly differentiated carcinomas (PDC). All cases were reviewed and re-classified according to the most recent WHO classification of thyroid tumors [26]. All PDC cases included in the study were defined according to the Turin proposal [27]. No oncocytic PDC was included in the study. In the groups of follicular and oncocytic carcinomas, 10 cases were re-classified as high-grade differentiated thyroid carcinomas (HG-DTC) because of the presence of tumor necrosis (7 cases) and/or mitotic index ≥ 5 in 2mm^2^ (4 cases, range of mitotic index from 5 to 9). For statistical purpose, these cases were included in the group of differentiated carcinomas (FC and OC) to keep separate PDC subtype based on its different and specific tumor architecture. However, for survival analyses, HG-DTC cases were also grouped together with PDC as the group of “high-grade follicular cell-derived thyroid carcinoma” (HG-FCDTC), to follow the most recent WHO classification scheme.

The following parameters were assessed and recorded: sex, age at diagnosis, tumor diameter, presence of tumor capsule, presence of capsular invasion, presence of vascular invasion, extent of vascular invasion (≥ or < 4 blood vessels involved), presence of necrosis, mitotic index in 2 mm^2^, status of the surgical margins and TNM stage according to AJCC 8th edition. The presence of tumor infiltrating lymphocytes (TILs) was recorded as absent, present “non-brisk” and present “brisk,” according to a previous study by our group in papillary thyroid carcinoma [18], that applied a proposal by the International Immuno-Oncology Biomarkers Working Group [28]. Briefly, TILs were evaluated assessing the average TILs in the complete tumor area (both central tumor and invasive margin) excluding TILs at a distance outside of the tumor borders or in tumor zones with necrosis and regressive changes. The percentage of stromal TILs was semi-quantitatively determined as the percentage of the stromal area that shows a dense mononuclear infiltrate. Since no recommendation of specific cut offs is available, we arbitrarily defined as “absent,” “present non-brisk,” and “present brisk” the presence of TILs corresponding to 0–5%, 6–20%, and > 20%, respectively. Follow-up information was available in 125 cases.

Before the study started, all cases were de-identified and coded by a pathology staff member not involved in the study, and all data were accessed anonymously. The study was approved by the local Ethical Committee (#610, date December 20th, 2017) and conducted in accordance with the principles set out in the Declaration of Helsinki. Considering the retrospective nature of this research protocol and that it had no impact on patients’ care, no specific written informed consent was required.

### Evaluation of intratumoral fibrosis

To evaluate intratumoral fibrosis, hematoxylin and eosin (H&E) slides of each case were reviewed independently by two investigators (EV and GVT). All available slides were evaluated for each case (from 1 to 32, mean 12). Fibrosclerotic changes were estimated evaluating all available sections at 4 × objective magnification, to assess in each field the percentage of fibrosis in the tumor area, to calculate the final percentage for each case. In selected cases (# 7), Masson’s trichrome and silver staining for reticulin were performed for determining the presence and extent of fibrosis.

**Fig. 1:**
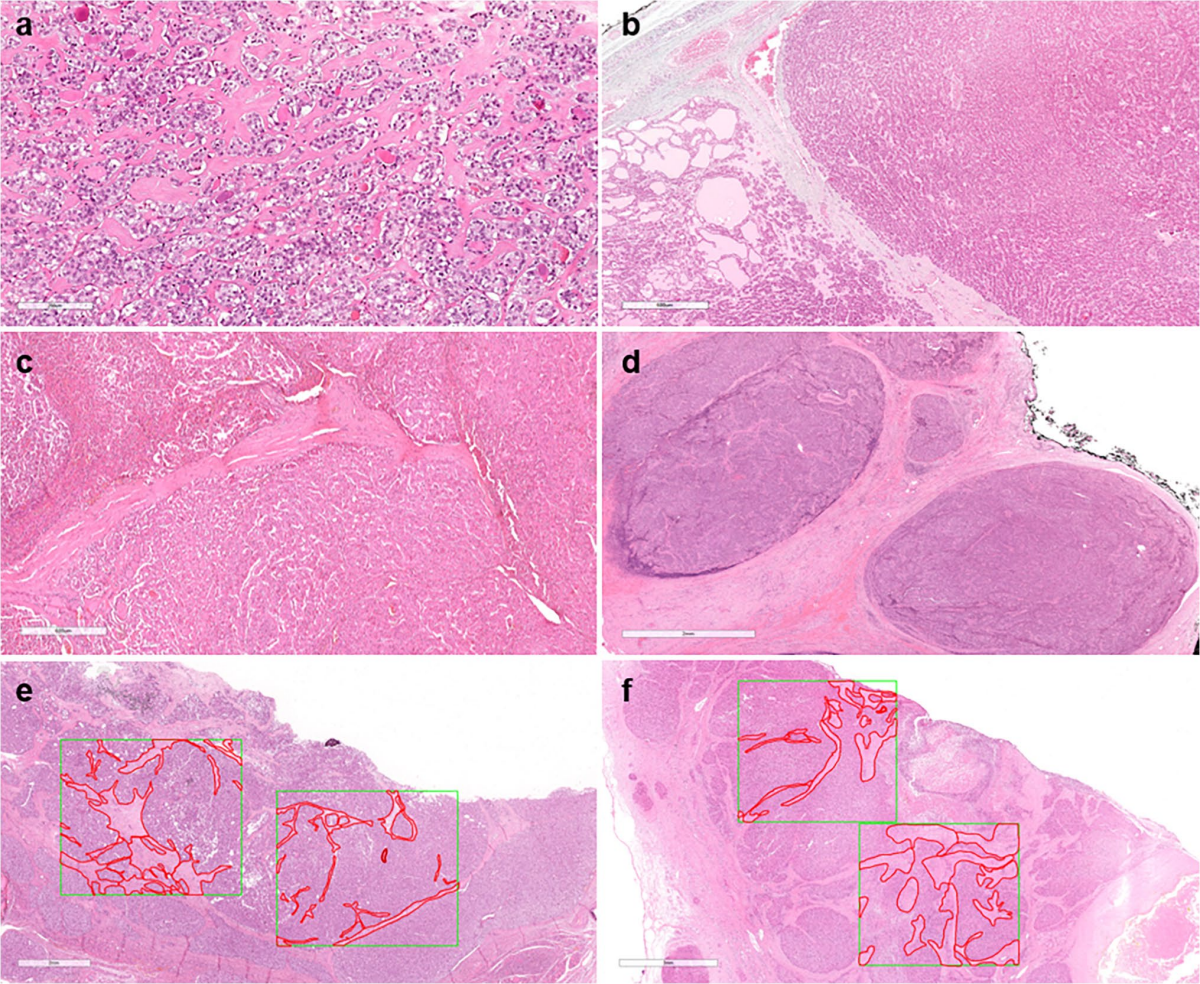
Representative images of intra-tumoral fibrosclerotic changes, occurring as inter-follicular fibrosis (**a** follicular carcinoma), inter-nodular stemming from the tumor capsule (**b** follicular carcinoma, **d**, poorly differentiated carcinoma), and intratumoral fibrosis (**c** oncocytic carcinoma). Digital imaging evaluation of fibrosclerotic changes areas, manually selected (red areas) and quantified using Aperio Scanner ScanScope XT tools (**e**, **f**)

Fibrosclerotic changes included generally thin inter-follicular fibrous tissue deposition or wide inter-nodular fibrous septa, these latter either intratumoral or stemming from the tumor capsule and penetrating into the lesion (Fig. 1a–d). Stromal edema and areas of fibrosis related to previous fine needle aspiration biopsies were excluded.

The cases were scored based on the percentage of tumor area with fibrosclerotic tissue and then sub-grouped into cases with absent = 0, mild = 1 (from 1 o to 10%), and extensive = 2 (> 10%) fibrosclerotic changes for statistical purposes. The type of fibrous tissue deposition (inter-follicular vs. inter-nodular) was not further considered for statistical purposes. All cases with a discordant result between the two observers, namely, those that were grouped differently, were evaluated in one session at a multi-head microscope by all participants to the study, to exclude biases due to the presence of unspecific stromal changes (i.e., edematous or artifactual changes) and to reassess the percentage of fibrosis in each discordant case, in blind of the previous scoring. The result obtained by the collegial revision was then used for statistical purposes. A selected series of 65 cases with a positive score at light microscopy for mild or extensive fibrosis was also analyzed using digital image analysis (on Aperio Scanner ScanScope XT; Leica Biosystems) testing the concordance between the estimation of the percentage of fibrosis per tumor area under light microscopy and an automated quantification by means of the digital image software Aperio ImageScope version 12.4 (Leica Biosystems). These cases were selected among the 78 cases with fibrosis, excluding those with a quality of H&E adequate for light microscopy evaluation, but inadequate for optimal digital microscope scanning (i.e., with alterations in terms of heterogeneity of thickness or staining artifacts that would have prevented optimal focusing of the scanner). For each case, 2 random fields of 20 mm^2^ were selected from one representative digitalized slide, and the area corresponding to fibrotic tissue in each field was identified and annotated manually by one of the investigators (GO) using the digital image tool (Fig. 1e, f), in blind of the scorings obtained by the two other observers. The annotated area was calculated in mm^2^ by the software, and its relative percentage was estimated. The total area under evaluation consisted of 40 mm^2^, and in most cases, it was at least representative of 50% of the tumor area in the evaluated slide. For digital image analysis, no specific protocols or pre-processing steps were applied.

### Statistical analysis

All the graphs, calculations, and statistical analyses were performed using GraphPad Prism software version 8.0 (GraphPad Software, San Diego, CA, USA). The differences in the distribution of variables were analyzed using parametric and non-parametric tests (Student’s *t* test, chi-square test, and Fisher’s exact test). The disease-free survival (DFS) was calculated from the date of surgical excision of the primary tumor to the date of the first relapse or last follow-up. Relapse was considered as the development of recurrence or distant metastases, and/or biochemical recurrence (> 2 ng/ml levels of thyroglobulin after TSH stimulation). Disease-specific survival (DSS) was calculated from the date of the surgical excision of the primary tumor to the date of death or last follow-up. Since 5 cases of FC and OC, only, died of the disease (including one case of HG-DTC), DSS analysis was restricted to PDC group. Survival curves between different groups were plotted using the Kaplan–Meier method, and the statistical comparisons were performed with Log-rank test. *p*-values < 0.05 were considered significant.

## Results

### Interobserver reproducibility of fibrosis evaluation

**Fig. 2:**
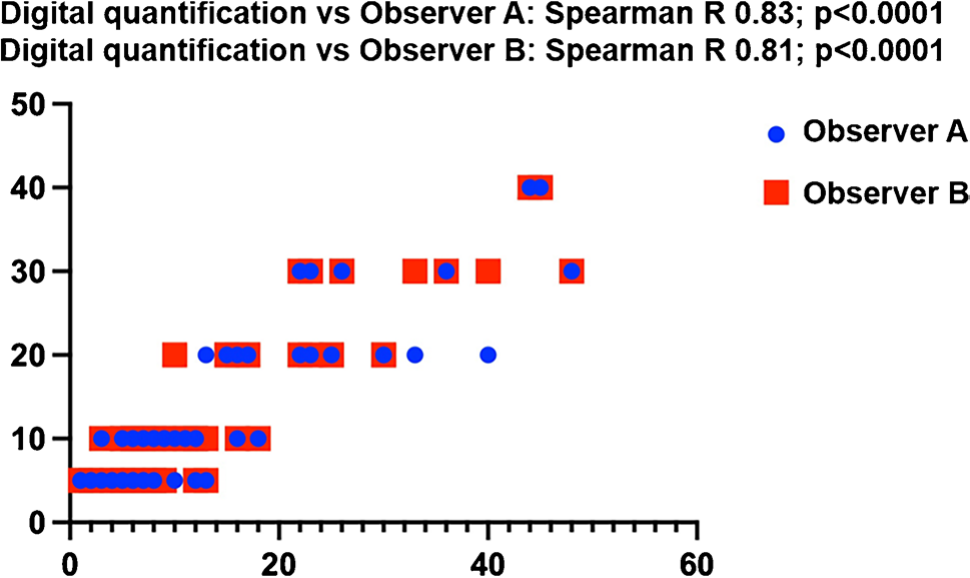
Spearman’s correlation between observers and digital imaging quantification

Two independent pathologists (EV and GVT) reviewed all H&E slides and scored the cases recording the percentage of tumor area with fibrosclerotic changes, irrespective of the type of fibrous tissue deposition. Spearman’s correlation between the two observers was very high, with a *R* value of 0.97 (*p* < 0.0001). The two pathologists were also both highly concordant with digital image quantification in the 65 cases analyzed with the Aperio Scanner ScanScope X (*R*: 0.83 and *R*: 0.82, both *p* values < 0.0001) (Fig. 2). Masson’s trichrome and silver-based reticulin stainings were used to ease the recognition of fibrous tissue deposition only in challenging cases, to better distinguish whether stromal changes consisted of edematous or desmoplastic stromal reaction. However, the added value of histochemistry appeared to be little; therefore, it was not applied more extensively and was not further considered for the statistical evaluations.

### Correlation between fibrosclerotic changes and clinico-pathological parameters

Main clinical and pathological associations are summarized in Table 1. In the whole series, fibrosclerotic changes were observed in 78 (59%) cases, being mild in 48 (36%) and extensive in 30 (23%) cases. The presence of fibrosis strongly correlated with histological type (*p* = 0.0002). It was more prevalent in PDC (38/48 cases, 79%) as compared to FC (28/53 cases, 53%) and OC (12/31 cases, 39%). Extensive fibrosis was predominant in PDC (20/38 cases, 53%), whereas it was restricted to 9/28 FC (32%) and to only 1/12 OC cases (8%). In the ten cases of HG-DTC, it was absent in three, mild in 6, but extensive in one case, only. Inter-follicular pattern was predominant in FC, whereas inter-nodular pattern was more prevalent in OC and PDC, although, in general, heterogeneous patterns coexisting within the same lesion were observed, and therefore, the quantity rather than the quality of fibrosclerotic changes was further considered for statistical purposes. TILs were absent in the vast majority of FC and OC cases and in about a third of PDC. Therefore, due to the low number of TILs-positive cases, this parameter was not further considered.

**Table 1:** Distribution of major clinical and pathological parameters according to the presence of fibrosclerotic changes

Parameter		Absent (#54)	Mild (≤ 10%) (#48)	Extensive (> 10%) (#30)	*p* value
Age	Mean (range)	52 (8–87)	61 (32–86)	66 (39–89)	0.0001
Sex	M/F	14/40	15/33	11/19	0.58
Histological type	FC^a^	25	19	9	0.0002
	OC^b^	19	11	1	
	PDC^c^	10	18	20	
Tumor size (cm)	< 2	9	4	16	0.003
	2–4	29	15	11	
	> 4	16	29	19	
Tumor capsule	Absent	2	5	7	0.02
	Present	52	43	23	
Capsular invasion^d^	Absent	18	11	1	0.02
	Present	34	32	22	
Vascular invasion	Absent	4	2	0	0.29
	Present	50	46	30	
Extent of vascular invasion^e^	< 4	27	12	6	0.002
	≥ 4	23	34	24	
Extent of vascular invasion (PDC excluded)	< 4	24	12	4	0.28
	≥ 4	16	16	6	
Necrosis	Absent	44	25	8	< 0.0001
	Present	10	23	22	
Mitotic index	Mean	1.2	2.1	2.7	0.0013
Surgical margins	R0	42	30	15	0.03
	R +	12	18	15	
pT stage	pT1-2	22	17	7	0.27
	pT3-4	32	31	23	
pT stage (PDC excluded)	pT1-2	31	16	3	0.043
	pT3-4	13	14	7	
pN stage	pN0	51	42	18	0.0001
	pN +	3	6	12	
Status^f^	NED^g^/DOC^h^	44	27	13	0.0003
	AWD^i^/DOD^j^	7	18	16	
Status (PDC excluded)	NED/DOC	38	21	6	0.074
	AWD/DOD	3	6	3	

^a^*FC*, follicular carcinoma; ^b^*OC*, oncocytic carcinoma; ^c^*PDC*, poorly differentiated carcinoma; ^d^14 cases with no capsule excluded; ^e^6 cases with no vascular invasion excluded; ^f^7 cases missing; ^g^*NED*, no evidence of disease; ^h^*DOC*, died other causes; ^i^*AWD*, alive with disease; ^j^*DOD*, died of disease

Presence and extent of fibrosclerotic changes was associated with age (*p* = 0.0001), partly but not exclusively as the consequence of the association with PDC histology that showed the highest mean age, as compared to the other histotypes (57, 50, and 64 years in FC, OC, and PDC, respectively, *p* = 0.0007; not shown in Table 1).

The presence of fibrosclerotic changes was also overall significantly associated with tumor size (*p* = 0.003) but it is worth to notice that intratumoral fibrosis was not exclusively observed in large tumors. Indeed, the majority of cases with a tumor size < 2 cm had extensive fibrosis. The absence of a tumor capsule was significantly associated with presence and extent of fibrosclerotic changes (*p* = 0.02). However, absence of a tumor capsule was observed in PDC cases, only, whereas all FC and OC cases were encapsulated, and no case showed criteria for “widely invasive” FC and OC. In encapsulated cases, fibrosclerotic changes occurred more frequently in cases associated to capsular invasion (*p* = 0.02). Presence and extent of vascular invasion was apparently not correlated with intratumoral fibrosis. Extensive (≥ 4 involved blood vessels) vascular invasion, only, was significantly associated with the presence of fibrosclerotic changes (*p* = 0.002), but failed to reach statistical significance once PDC cases were excluded. The presence of necrosis was strongly associated with the presence and extent of fibrosis (*p* < 0.0001), possibly as the result of the concurrent association with PDC type. Mitotic index (as a mean value and not using cut-offs for PDC or high grade differentiated thyroid carcinoma) was also significantly associated with the presence of intratumoral fibrosis (*p* = 0.0013), but failed to reach statistical significance when PDC were excluded from the analysis (*p* = 0.399, not shown in Table 1). Positive resection margins and high pT stage were also associated with the presence of fibrosis. In particular, the latter was not statistically significant in the whole series but was significant (*p* = 0.043) in the group of FC and OC. Finally, a strong statistical significance was observed between the presence and extent of fibrosclerotic changes and positive nodal status (*p* = 0.0001) and adverse clinical outcome (*p* = 0.0001). Despite the low number of events, the association between presence of intratumoral fibrosis and aggressive clinical course maintained a trend to statistical significance also when PDC cases were excluded from the analysis (*p* = 0.07).

### Survival analyses

Mean follow-up times were 9.4 years for the whole series (range 0.3–25), 10.3 years for the series of FC and OC (including the 6 cases of HG-DTC with follow up information available; range 0.3–21), and 7.7 years for the PDC series (range 0.6–25). The prognostic impact of the presence of fibrosclerotic changes was investigated in terms of both disease-free survival and disease-specific survival, in parallel with main known prognostic parameters in non-papillary, non-anaplastic thyroid carcinomas, such as age, extent of vascular invasion, pTN stage, and status of the resection margins (Table 2). Out of 77 FC and OC cases having follow-up information available, 12 cases (16%) had disease relapse and 5 cases (6%, including one HG-DTC) died of their disease. In PDC cases, all with available follow-up information, 29 cases (60%) had disease relapse and 17 cases (35%) died of their disease. Multivariate analysis in the whole series was not performed due to the limited number of events, but the potential influence of histological type in survival analyses was eliminated separately testing candidate prognostic variables in FC, OC, and PDC groups.

**Table 2:** Univariate survival analyses

**Disease-free survival analysis**						
Parameter	All cases (# 126)		FC and OC, only (#77)		PDC, only (#48)	
	HR^a^ [CI^b^]	*p*	HR [CI]	*p*	HR [CI]	*p*
Age (> 55 vs ≤ 55)	2.84 [1.53–5.3]	0.0037	12.36 [3.76–40.5]	0.002	0.68 [0.28–1.67]	0.34
PDC vs FC + OC	5.2 [2.70–10.02]	< 0.0001	/	/	/	/
OC vs. FC	/	/	0.14 [0.04–0.45]	0.026	/	/
Fibrosis (0 vs. 1–2)	0.21 [0.11–0.38]	< 0.0001	0.17 [0.05–0.55]	0.009	0.52 [0.22–1.24]	0.22
Extent of vascular invasion (≥ 4 vs. < 4)	3.49 [1.85–6.57]	0.001	3.04 [0.86–10.7]	0.083	Not tested (4 cases with < 4 positive vessels, only)	
pT3-4 vs. pT1-2	4.98 [2.68–9.26]	< 0.0001	8.12 [0.86–10.7]	0.001	1.00 [0.38–2.63]	0.99
pN + vs. pN0	4.82 [1.79–12.94]	< 0.0001	Not tested (3 cases with pN + , only)		2.38 [1.03–5.45]	0.041
Resection margins (R1 vs. R0)	1.78 [0.90–3.52]	0.067	1.52 [0.34–6.7]	0.53	0.98 [0.47–2.05]	0.98
**Disease-specific survival analysis**						
Parameter	All cases (# 126)		FC and OC, only (#77)		PDC, only (#48)	
	HR [CI]	*p*	HR [CI]	*p*	HR [CI]	*p*
Age (> 55 vs. ≤ 55)	3.3 [1.42–7.69]	0.019	Not tested (5 cases died of disease, only)		0.98 [0.32–3.07]	0.98
PDC vs. FC + OC	5.84 [2.46–13.9]	< 0.0001			/	/
Fibrosis (0 vs. 1–2)	0.05 [0.02–0.13]	< 0.0001			0.27 [0.08–0.86]	0.027
Extent of vascular invasion (≥ 4 vs. < 4)	3.44 [1.48–7.99]	0.014			Not tested (4 cases with < 4 positive vessels, only)	
pT3-4 vs. pT1-2	5.64 [2.44–13.02]	0.001			1.49 [0.42–5.30]	0.54
pN + vs. pN0	4.88 [1.37–17.33]	< 0.0001			1.73 [0.59–5.00]	0.31
Resection margins (R1 vs. R0)	1.88 [0.78–4.53]	0.11			1.08 [0.40–2.88]	0.88

^a^*HR*, hazard ratio; ^b^*CI*, confidential intervals

**Fig. 3:**
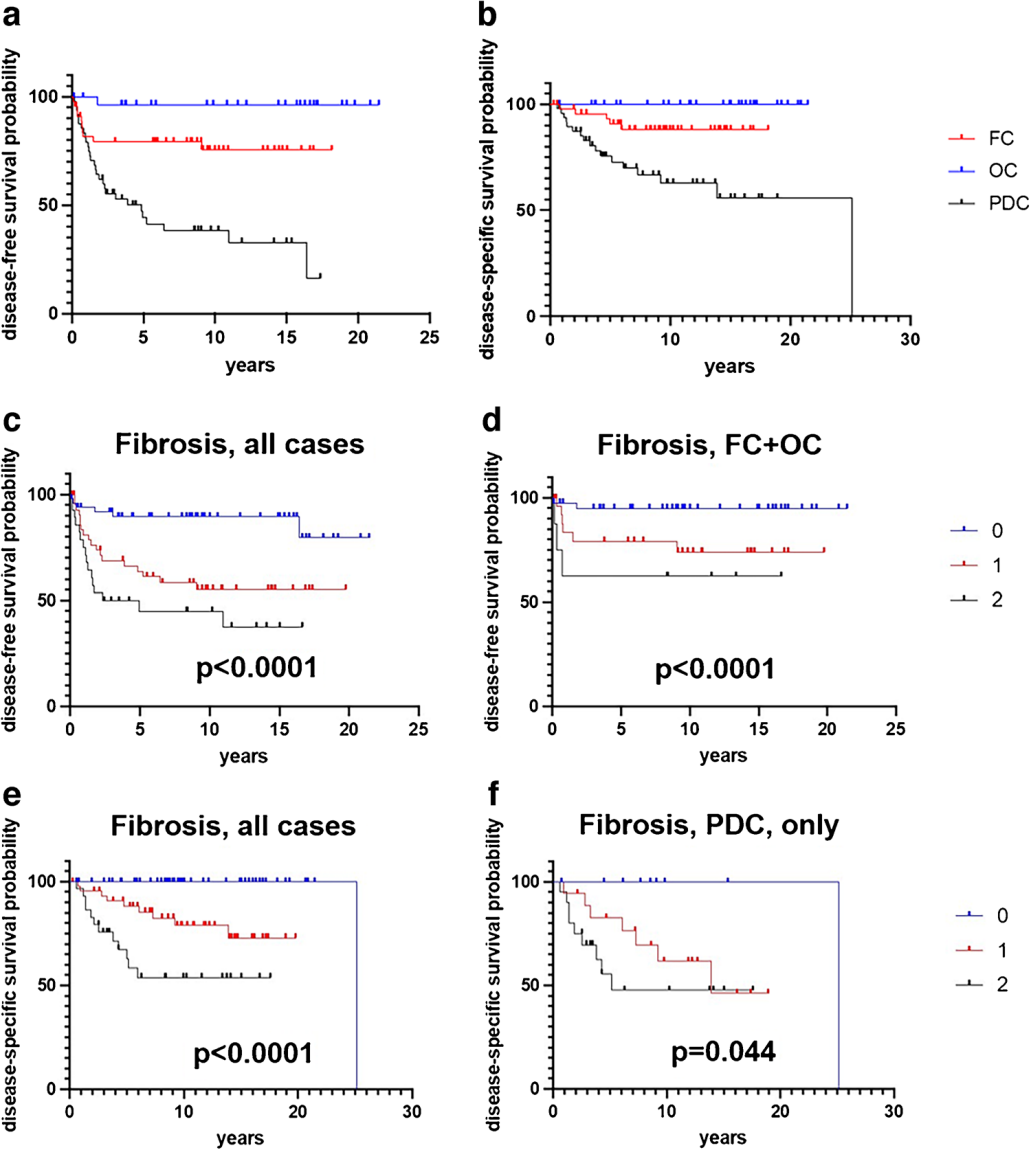
Kaplan–Meier estimates of disease-free and disease-specific survivals according to tumor histotype (*p* < 0.0001 and *p* = 0.0001, respectively) (**a**, **b**). Kaplan–Meier estimates of disease-free and disease-specific survivals according to the presence and extent of fibrosclerotic changes, in the whole series and in histological type-based subgroup analyses. Fibrosclerotic changes scores are: 0, absent; 1, present mild; 2, present extensive (**c**–**f**) (FC: follicular carcinoma; OC: oncocytic carcinoma; PDC: poorly differentiated carcinoma)

In our series, age > 55 years, extensive vascular invasion (≥ 4 involved blood vessels), high pT stage and positive nodal status were all parameters associated with shorter survival in both disease-free and disease-specific survival analyses. Positive resection margin status, although with a trend to significance, failed to show a statistically proven association with poorer prognosis. Histological PDC subtype was a strong indicator of poor prognosis in both disease-free and disease-specific survival analyses (Fig. 3a, b). The same result was observed comparing the whole group of HG-FCDTC (thus including HG-DTC and PDC) with FC and OC (Supplementary Fig. 1), with hazard ratio values of 5.36 [confidential intervals: 2.84–10.13; *p* < 0.0001] and 2.30 [confidential intervals: 1.11–4.76; *p*: 0.015] at disease-free and disease-specific survival analyses, respectively. Interestingly, in the group of differentiated thyroid carcinomas, OC cases showed in disease-free survival analysis a better survival as compared to FC, either including (Table 2) or excluding (Supplementary Table 1) HG-DTC cases.

The association between fibrosclerotic changes and adverse clinical outcome described above was strongly confirmed by univariate survival analyses. In fact, cases with absence, presence of mild or extensive fibrosclerotic changes were associated with progressively poorer survival in both disease-free and disease-specific survival analyses (Fig. 3c, e). Log-rank test in both models confirmed the lower risk for events in cases with absence as compared to cases with presence of intratumoral fibrosis (irrespective of its extent). Notably, statistical significance of fibrosclerotic changes was retained in disease-free survival analysis in the group of FC and OC (thus excluding PDC) (Fig. 3d), as also confirmed by log rank test together with age and pT stage. The same results were obtained also excluding the 6 cases of HG-DTC (Supplementary Table 1). Subgroup analysis of PDC cases or the whole group of HG-FCDTC was limited by the small number of cases. Even with this limitation, although with no statistical significance in disease-free survival analysis, fibrosclerotic changes were associated with adverse prognosis in disease-specific survival, as also confirmed by log rank test showing fibrosclerotic changes as the single parameter associated with statistical significance in both PDC subtype alone (Table 2, Fig. 3f) and in the whole group of HG-FCDTC (Supplementary Table 1).

## Discussion

In the present study, the aim was to assess the presence and prognostic value of intratumoral fibrosis in non-papillary, non-anaplastic follicular cell-derived thyroid carcinomas.

Intratumoral fibrosis has been shown in different studies to be associated with aggressive disease characteristics in papillary carcinoma [18, 29] and in medullary carcinoma [30, 31], but it was never investigated in details in FC, OC, or HG-FCDTC, so far.

Fibrosclerotic changes were observed more frequently in PDC, but a relevant proportion of FC and OC cases, including HG-DTC, also had intratumoral fibrosis, although predominantly mild. Intratumoral fibrosis shared different patterns in the different histological types, probably as a consequence of the tumor structures. In fact, FC showed a predominant inter-follicular pattern, whereas PDC and OC showed a predominant inter-nodular pattern in the presence of a more solid/trabecular or large nesting architecture. However, several cases had mixed patterns of intratumoral fibrosis and the pattern itself, irrespective of the extent of fibrosclerotic changes, failed to show statistical significance in preliminary analyses (data not shown). In selected cases, we added to conventional H&E special histochemical stains, to highlight the tumor architecture and the presence of fibrous tissue in cases of hard interpretation, but they did not represent an added value, and were not subsequently employed.

Another aim of the study was to assess the interobserver reproducibility of fibrosclerotic changes evaluation, to support its potential use as an additional morphological parameter to be included in the characterization of the thyroid cancer histotypes here investigated. The percentage of intratumoral fibrosis per tumor area was recorded by two independent investigators, and the correlation between them was almost perfect. Moreover, in selected cases, we added digital imaging quantification of intratumoral fibrosis, and the investigators strongly agreed with automated quantitative evaluation. Overall, these data show that the evaluation of the presence and extent of intratumoral fibrosis is an easy to assess and reproducible tool in the pathological work-up of thyroid carcinoma.

The presence of fibrosclerotic changes was scored as absent, mild, or extensive for the statistical comparisons and to design a simple categorization. Although this simplified approach could have caused loss of information, it met the need of an easy semi-quantitative scoring system for clinical purposes adding a novel possible information on patient prognosis.

The presence of fibrosclerotic changes clearly characterized cases with aggressive pathological features, such as PDC subtype of HG-FCDTC, presence of necrosis, increased mitotic index, positive nodal status, and positive resection margins. By contrast, intratumoral fibrosis was not significantly correlated with either tumor pT stage or tumor size. Concerning the latter, it proved to be statistically significant overall, but extensive fibrosclerotic changes were observed even in cases with a size < 2 cm. Invasive properties of tumors were differently associated with intratumoral fibrosis. In fact, the presence and extent of fibrosclerotic changes correlated with the presence of capsular invasion, whereas vascular invasion was not associated with it, except for extensive (≥ 4 blood vessels involved) angioinvasive PDC cases. Finally, aggressive clinical course, in terms of tumor relapse with or without disease-related deaths, was significantly associated with intratumoral fibrosis, with 34/41 aggressive disease cases (83%) having fibrosclerotic changes, as compared to 44/84 (52%) cases associated to a clinically indolent disease. Nonetheless, most of the associations described above are possibly biased by the more common occurrence of fibrosclerotic changes in PDC histotype, that is characterized by a more aggressive pathological phenotype. Taking this into consideration, survival analyses planned to weight the prognostic impact of intratumoral fibrosis were conducted, whenever possible, both in the whole series and separately in PDC and FC plus OC subgroups. Sub-analysis comparing the whole group of HG-FCDT (PDC plus HG-DTC) to FC and OC was also conducted. This approach helped to exclude possible histotype-related biases, although it caused a decrease in the potency of statistical analyses due to the lower number of included cases.

Reliability of survival models was supported by the fact that almost all know prognostic factors we included in the analyses (age, histological type, extent of vascular invasion and pTN stage) showed a statistical significance, in both disease-free and disease-specific survival tests. Noteworthy, fibrosclerotic changes (comparing absence vs. presence, irrespective of its extent) were among the strongest statistically significant factors influencing prognosis in both disease-free and disease-specific survival models. Subgroup analysis of disease-free survival in FC and OC, only—either including or excluding HG-DTC cases—confirmed the strong association of the presence of fibrosclerotic changes with an increased risk of tumor relapse, whereas in disease-specific survival analysis of PDC subtype and in the whole group of HG-FCDTC, fibrosclerotic changes were associated with an increased risk of disease-associated death.

Intratumoral fibrosis and cancer are closely related. More or less extensive desmoplastic stroma is a feature of several carcinomas, including breast, pulmonary, and pancreatic ones, and it was found to be associated to tumor progression [32]. Intratumoral fibrosis results from the deposition of a cross-linked collagen matrix by cancer-associated fibroblasts (CAFs). This type of fibrosis affects intratumoral immunity and may influence metastatic spread, since CAFs and tumor cells are regulated by provisional matrix molecules that interfere with stromal collagen cross-link, favoring metastases [33]. In thyroid cancer, tumor micro-environment (TME) is composed of a plethora of various cell types: stromal cells, immune cells, endothelial cells, and pericytes can be found in the environment together with CAFs. All these cells coexist and continuously interact with thyroid cancer cells, supporting tumor growth and metabolism, promoting angiogenesis and cancer progression [34]. Considering these observations, TME of thyroid cancer is a rich landscape for future studies, in particular regarding the possibility to develop novel therapies targeting CAFs [35]. It was out of the scopes of the paper to deeply characterize the tumor microenvironment together with the assessment of tumor fibrosis. However, we could observe that fibrosclerotic changes were not significantly associated with the presence of TILs, with special reference to FC and OC (data not shown). In general terms, this observation is in line with previous gene expression studies focusing on immune-related gene signatures showing that PDC have a “cold” immune micro-environment, as compared to papillary and anaplastic carcinomas [36]. Similarly, cancer-associated fibroblasts may be responsible for or co-operate in the development of intratumoral fibrosis in thyroid cancer [34]. However, available data on the role of CAF in thyroid cancer and on their potential role as biomarkers are limited to papillary and anaplastic carcinoma models. A single study included also a small set of PDC reported the expression of CAF-associated markers (collagen type 1 gene, *COL1A1*, and alpha smooth muscle actin gene, *ACTA2*) in such cases, similar to papillary and anaplastic carcinomas [37]. These studies are missing in the literature for FC and OC, and the present series would deserve future investigation at the molecular level to more deeply depict whether the presence of intratumoral fibrosis may reflect specific tumor microenvironment characteristics.

In conclusions, intratumoral fibrosclerotic changes represent a novel potential prognostic factor in non-papillary, non-anaplastic, follicular cell-derived thyroid carcinomas (follicular, oncocytic and HG-FCDTC, with special reference to its PDC subtype). Fibrosclerosis is significantly associated with the presence of parameters of aggressiveness, and with decreased disease-free and disease specific survival rates, independently from the tumor histotype. The evaluation of intratumoral fibrosis is also easily assessable both manually and with the aid of digitalization system, with a very high interobserver agreement, supporting its potential use in the clinical diagnostic practice.

Supplementary Fig. 1 Kaplan Meier estimates of disease-free (left) and disease-specific (right) survivals (p < 0.0001 and p = 0.008, respectively) according to tumor histotype, incorporating PDC and high-grade differentiated carcinoma cases into the group of high-grade follicular cell-derived carcinoma (HG-FCDTC) (FC: follicular carcinoma; OC: oncocytic carcinoma; PDC: poorly differentiated carcinoma)

## Supplementary Information

Below is the link to the electronic supplementary material.

Supplementary file1 (DOCX 16 KB)

Supplementary file2 (TIF 1087 KB)

## Data Availability

The data that support the findings of this study are not openly available due to reasons of sensitivity and are available from the corresponding author upon reasonable request. Data are located in controlled access data storage at the Department of Oncology, University of Turin.

## References

[1] Zhang T, He L, Wang Z, Dong W, Sun W, Zhang P, Zhang H (2023) Risk factors for death of follicular thyroid carcinoma: a systematic review and meta-analysis. Endocrine 82:457–466. 10.1007/s12020-023-03466-9 37804444 10.1007/s12020-023-03466-9 PMC10618390

[2] Stegenga MT, van Velsen EFS, Oudijk L, Verburg FA, van Ginhoven TM, Peeters RP, Medici M, Visser WE, van Kemenade FJ (2024) Clinical and histopathological risk factors for radioactive iodine refractory follicular and oncocytic thyroid carcinoma. J Clin Endocrinol Metab. Feb 13:dgae084. 10.1210/clinem/dgae084 10.1210/clinem/dgae084 PMC11570392 38349206

[3] Leong D, Gill AJ, Turchini J, Waller M, Clifton-Bligh R, Glover A, Sywak M, Sidhu S (2023) The prognostic impact of extent of vascular invasion in follicular thyroid carcinoma. World J Surg 47:412–420. 10.1007/s00268-022-06696-6 36031639 10.1007/s00268-022-06696-6

[4] Ito Y, Hirokawa M, Masuoka H, Higashiyama T, Kihara M, Onoda N, Miya A, Miyauchi A (2022) Prognostic factors for follicular thyroid carcinoma: the importance of vascular invasion. Endocr J 69:1149–1156. 10.1507/endocrj.EJ22-0077 35491160 10.1507/endocrj.EJ22-0077

[5] Yamazaki H, Sugino K, Katoh R, Matsuzu K, Kitagawa W, Nagahama M, Rino Y, Saito A, Ito K (2023) Role of the degree of vascular invasion in predicting prognosis of follicular thyroid carcinoma. J Clin Endocrinol Metab 109:1291–1300. 10.1210/clinem/dgad689 10.1210/clinem/dgad689 38006314

[6] Jin M, Kim ES, Kim BH, Kim HK, Kang YE, Jeon MJ, Kim TY, Kang HC, Kim WB, Shong YK, Kim M, Kim WG (2021) Clinicopathological characteristics and disease-free survival in patients with Hürthle cell carcinoma: a multicenter cohort study in South Korea. Endocrinol Metab (Seoul) 36:1078–1085. 10.3803/EnM.2021.1151 34731935 10.3803/EnM.2021.1151 PMC8566133

[7] Matsuura D, Yuan A, Wang L, Ranganath R, Adilbay D, Harries V, Patel S, Tuttle M, Xu B, Ghossein R, Ganly I (2022) Follicular and Hurthle cell carcinoma: comparison of clinicopathological features and clinical outcomes. Thyroid 32:245–254. 10.1089/thy.2021.0424 35078345 10.1089/thy.2021.0424 PMC9206490

[8] Ghossein RA, Hiltzik DH, Carlson DL, Patel S, Shaha A, Shah JP, Tuttle RM, Singh B (2006) Prognostic factors of recurrence in encapsulated Hurthle cell carcinoma of the thyroid gland: a clinicopathologic study of 50 cases. Cancer 106:1669–1676. 10.1002/cncr.21825 16534796 10.1002/cncr.21825

[9] Humphreys BM, Memeh KO, Funkhouser A, Vaghaiwalla TM (2022) Prognostic factors and survival analysis of Hurthle cell carcinoma: a population-based study. Surgery 172:1379–1384. 10.1016/j.surg.2022.07.007 36038373 10.1016/j.surg.2022.07.007

[10] Wong KS, Lorch JH, Alexander EK, Marqusee E, Cho NL, Nehs MA, Doherty GM, Barletta JA (2019) Prognostic significance of extent of invasion in poorly differentiated thyroid carcinoma. Thyroid 29:1255–1261. 10.1089/thy.2019.0263 31397224 10.1089/thy.2019.0263

[11] Gubbiotti MA, Andrianus S, Sakhi R, Zhang Q, Montone K, Jalaly JB, Baloch Z (2023) Does the presence of capsule influence prognosis in poorly differentiated thyroid carcinoma? Hum Pathol 136:96–104. 10.1016/j.humpath.2023.04.005 37054782 10.1016/j.humpath.2023.04.005

[12] Asioli S, Erickson LA, Righi A, Jin L, Volante M, Jenkins S, Papotti M, Bussolati G, Lloyd RV (2010) Poorly differentiated carcinoma of the thyroid: validation of the Turin proposal and analysis of IMP3 expression. Mod Pathol 23:1269–1278. 10.1038/modpathol.2010.117 20562850 10.1038/modpathol.2010.117

[13] Xu B, Lubin DJ, Dogan S, Ghossein RA, Viswanathan K (2023) Significance of oncocytic features in poorly differentiated thyroid carcinoma — a bi-institutional experience. Virchows Arch 482:479–491. 10.1007/s00428-022-03422-4 36346459 10.1007/s00428-022-03422-4 PMC12606509

[14] Nicolson NG, Murtha TD, Dong W, Paulsson JO, Choi J, Barbieri AL, Brown TC, Kunstman JW, Larsson C, Prasad ML, Korah R, Lifton RP, Juhlin CC, Carling T (2018) Comprehensive genetic analysis of follicular thyroid carcinoma predicts prognosis independent of histology. J Clin Endocrinol Metab 103:2640–2650. 10.1210/jc.2018-00277 29726952 10.1210/jc.2018-00277

[15] Park H, Shin HC, Yang H, Heo J, Ki CS, Kim HS, Kim JH, Hahn SY, Chung YJ, Kim SW, Chung JH, Oh YL, Kim TH (2022) Molecular classification of follicular thyroid carcinoma based on TERT promoter mutations. Mod Pathol 35:186–192. 10.1038/s41379-021-00907-6 34497362 10.1038/s41379-021-00907-6 PMC8786663

[16] Yang H, Park H, Ryu HJ, Heo J, Kim JS, Oh YL, Choe JH, Kim JH, Kim JS, Jang HW, Kim TH, Kim SW, Chung JH (2022) Frequency of TERT promoter mutations in real-world analysis of 2,092 thyroid carcinoma patients. Endocrinol Metab (Seoul) 37:652–663. 10.3803/EnM.2022.1477 35864728 10.3803/EnM.2022.1477 PMC9449103

[17] Hescot S, Al Ghuzlan A, Henry T, Sheikh-Alard H, Lamartina L, Borget I, Hadoux J, Baudin E, Dupuy C, Nikitski AV, Nikiforov YE, Schlumberger M, Nikiforova MN, Leboulleux S (2022) Prognostic of recurrence and survival in poorly differentiated thyroid cancer. Endocr Relat Cancer 29:625–634. 10.1530/ERC-22-0151 36040800 10.1530/ERC-22-0151

[18] Metovic J, Cabutti F, Osella-Abate S, Orlando G, Tampieri C, Napoli F, Maletta F, Daniele L, Volante M, Papotti M (2023) Clinical and pathological features and gene expression profiles of clinically aggressive papillary thyroid carcinomas. Endocr Pathol 34:298–310. 10.1007/s12022-023-09769-x 37208504 10.1007/s12022-023-09769-x PMC10511602

[19] Xu B, Tuttle RM, Sabra MM, Ganly I, Ghossein R (2017) Primary thyroid carcinoma with low-risk histology and distant metastases: clinicopathologic and molecular characteristics. Thyroid 27:632–640. 10.1089/thy.2016.0582 28049366 10.1089/thy.2016.0582 PMC5421603

[20] Takeda M, Mikami T, Numata Y, Okamoto M, Okayasu I (2013) Papillary thyroid carcinoma with heterotopic ossification is a special subtype with extensive progression. Am J Clin Pathol 139:587–598. 10.1309/AJCPQZQN50HKIAHA 23596110 10.1309/AJCPQZQN50HKIAHA

[21] Koperek O, Akin E, Asari R, Niederle B, Neuhold N (2013) Expression of hypoxia-inducible factor 1 alpha in papillary thyroid carcinoma is associated with desmoplastic stromal reaction and lymph node metastasis. Virchows Arch 463:795–802. 10.1007/s00428-013-1484-3 24197448 10.1007/s00428-013-1484-3

[22] Rivera M, Ricarte-Filho J, Patel S, Tuttle M, Shaha A, Shah JP, Fagin JA, Ghossein RA (2010) Encapsulated thyroid tumors of follicular cell origin with high grade features (high mitotic rate/tumor necrosis): a clinicopathologic and molecular study. Hum Pathol 41:172–180. 10.1016/j.humpath.2009.08.011 19913280 10.1016/j.humpath.2009.08.011 PMC4573458

[23] Collini P, Sampietro G, Pilotti S (2004) Extensive vascular invasion is a marker of risk of relapse in encapsulated non-Hürthle cell follicular carcinoma of the thyroid gland: a clinicopathological study of 18 consecutive cases from a single institution with a 11-year median follow-up. Histopathology 44:35–39. 10.1111/j.1365-2559.2004.01729.x 14717667 10.1111/j.1365-2559.2004.01729.x

[24] Lang W, Choritz H, Hundeshagen H (1986) Risk factors in follicular thyroid carcinomas. A retrospective follow-up study covering a 14-year period with emphasis on morphological findings. Am J Surg Pathol 10:246–255 3706611

[25] Cracolici V, Kadri S, Ritterhouse LL, Segal JP, Wanjari P, Cipriani NA (2019) Clinicopathologic and molecular features of metastatic follicular thyroid carcinoma in patients presenting with a thyroid nodule versus a distant metastasis. Am J Surg Pathol 43:514–522. 10.1097/PAS.0000000000001208 30557173 10.1097/PAS.0000000000001208

[26] Baloch ZW, Asa SL, Barletta JA, Ghossein RA, Juhlin CC, Jung CK, LiVolsi VA, Papotti MG, Sobrinho-Simões M, Tallini G, Mete O (2022) Overview of the 2022 WHO classification of thyroid neoplasms. Endocr Pathol 33:27–63. 10.1007/s12022-022-09707-3 35288841 10.1007/s12022-022-09707-3

[27] Volante M, Collini P, Nikiforov YE, Sakamoto A, Kakudo K, Katoh R, Lloyd RV, LiVolsi VA, Papotti M, Sobrinho-Simoes M, Bussolati G, Rosai J (2007) Poorly differentiated thyroid carcinoma: the Turin proposal for the use of uniform diagnostic criteria and an algorithmic diagnostic approach. Am J Surg Pathol 31:1256–1264. 10.1097/PAS.0b013e3180309e6a 17667551 10.1097/PAS.0b013e3180309e6a

[28] Hendry S, Salgado R, Gevaert T et al (2017) Assessing tumor-infiltrating lymphocytes in solid tumors: a practical review for pathologists and proposal for a standardized method from the international immunooncology biomarkers working group: part 1: assessing the host immune response, TILs in invasive breast carcinoma and ductal carcinoma in situ, metastatic tumor deposits and areas for further research. Adv Anat Pathol 24:235–251. 10.1097/PAP.0000000000000162 28777142 10.1097/PAP.0000000000000162 PMC5564448

[29] Wang HQ, Li Y, Song X, Ma YQ, Li JL, Li YX, Wang GF, Liu P, Liu PL, Cao S, Shi HY (2022) Significance of interstitial fibrosis and p16 in papillary thyroid carcinoma. Endocr J 69:1253–1259. 10.1507/endocrj.EJ22-0010 35718445 10.1507/endocrj.EJ22-0010

[30] Moura MM, Cabrera RA, Esteves S, Cavaco BM, Soares P, Leite V (2021) Correlation of molecular data with histopathological and clinical features in a series of 66 patients with medullary thyroid carcinoma. J Endocrinol Invest 44:1837–1846. 10.1007/s40618-020-01456-6 33575974 10.1007/s40618-020-01456-6

[31] Scheuba C, Kaserer K, Kaczirek K, Asari R, Niederle B (2006) Desmoplastic stromal reaction in medullary thyroid cancer - an intraoperative “marker” for lymph node metastases. World J Surg 30:853–859. 10.1007/s00268-005-0391-4 16680600 10.1007/s00268-005-0391-4

[32] Piersma B, Hayward MK, Weaver VM (2020) Fibrosis and cancer: a strained relationship. Biochim Biophys Acta Rev Cancer 1873:188356. 10.1016/j.bbcan.2020.188356 32147542 10.1016/j.bbcan.2020.188356 PMC7733542

[33] Meng Q, Luo X, Chen J, Wang D, Chen E, Zhang W, Zhang G, Zhou W, Xu J, Song Z (2020) Unmasking carcinoma-associated fibroblasts: key transformation player within the tumor microenvironment. Biochim Biophys Acta Rev Cancer 1874:188443. 10.1016/j.bbcan.2020.188443 33035642 10.1016/j.bbcan.2020.188443

[34] Shin E, Koo JS (2022) Cell component and function of tumor microenvironment in thyroid cancer. Int J Mol Sci 23:12578. 10.3390/ijms232012578 36293435 10.3390/ijms232012578 PMC9604510

[35] Tahara M, Schlumberger M, Elisei R, Habra MA, Kiyota N, Paschke R, Dutcus CE, Hihara T, McGrath S, Matijevic M, Kadowaki T, Funahashi Y, Sherman SI (2017) Exploratory analysis of biomarkers associated with clinical outcomes from the study of lenvatinib in differentiated cancer of the thyroid. Eur J Cancer 75:213–221. 10.1016/j.ejca.2017.01.013 28237867 10.1016/j.ejca.2017.01.013

[36] Giannini R, Moretti S, Ugolini C, Macerola E, Menicali E, Nucci N, Morelli S, Colella R, Mandarano M, Sidoni A, Panfili M, Basolo F, Puxeddu E (2019) Immune profiling of thyroid carcinomas suggests the existence of two major phenotypes: an ATC-like and a PDTC-like. J Clin Endocrinol Metab 104:3557–3575. 10.1210/jc.2018-01167 30882858 10.1210/jc.2018-01167

[37] Minna E, Brich S, Todoerti K, Pilotti S, Collini P, Bonaldi E, Romeo P, Cecco L, Dugo M, Perrone F, Busico A, Vingiani A, Bersani I, Anichini A, Mortarini R, Neri A, Pruneri G, Greco A, Borrello MG (2020) Cancer associated fibroblasts and senescent thyroid cells in the invasive front of thyroid carcinoma. Cancers (Basel) 12:112. 10.3390/cancers12010112 31906302 10.3390/cancers12010112 PMC7016563

