# Supplementary material for: Prognostic impact of fibrosclerotic changes in non-papillary, non-anaplastic, follicular cell-derived thyroid carcinomas
Source: Virchows Arch. 2025 Jan 23;487(3):701–11. doi: 10.1007/s00428-025-04028-2 (PMC12488736; doi:10.1007/s00428-025-04028-2)
Supplement: Supplementary file 1 — Supplementary file1 (DOCX 16 KB) [file 428_2025_4028_MOESM1_ESM.docx]

**Supplementary Table 1.** Univariate survival analyses.

| **Disease-free survival analysis** | | | | |
| --- | --- | --- | --- | --- |
| Parameter | FC and OC, only (#71) | | HG-FCDTC, only (#54) | |
|  | HR [CI] | p | HR [CI] | p |
| Age (>55 vs <55) | 12.3 [3.51-43.14] | 0.022 | 0.51 [0.21-1.24] | 0.07 |
| OC vs FC | 0.15 [0.04-0.54] | 0.041 | / | / |
| Fibrosis (0 vs 1-2) | 0.18 [0.05-0.63] | 0.014 | 0.54 [0.23-1.28] | 0.24 |
| Extent of vascular invasion (>4 vs <4) | 1.64 [0.44-6.10] | 0.47 | 1.23 [0.46-3.26] | 0.69 |
| pT3-4 vs pT1-2 | 5.42 [1.55-19] | 0.015 | 1.14 [0.48-2.69] | 0.76 |
| pN+ vs pN0 | Not tested (3 cases with pN+, only) | | 2.07 [0.93-4.62] | 0.036 |
| Resection margins (R1 vs R0) | 1.67 [0.36-7.8] | 0.45 | 0.94 [0.46-1.90] | 0.85 |
| **Disease-specific survival analysis** | | | | |
| Parameter | FC and OC, only (#71) | | HG-FCDTC, only (#54) | |
|  | HR [CI] | p | HR [CI] | p |
| Age (>55 vs <55) | Not tested (4 cases died of disease, only) | | 0.85 [0.29-2.48] | 0.98 |
| Fibrosis (0 vs 1-2) |  |  | 0.17 [0.06-0.49] | 0.031 |
| Extent of vascular invasion (>4 vs <4) |  |  | 1.03 [0.30-3.51] | 0.95 |
| pT3-4 vs pT1-2 |  |  | 1.28 [0.41-4.03] | 0.69 |
| pN+ vs pN0 |  |  | 1.62 [0.58-4.53] | 0.30 |
| Resection margins (R1 vs R0) |  |  | 1.02 [0.41-2.58] | 0.95 |

^a^HR: hazard ratio; ^b^CI: confidential intervals
