# Supplementary figures and images for: Prognostic impact of fibrosclerotic changes in non-papillary, non-anaplastic, follicular cell-derived thyroid carcinomas
Source: Virchows Arch. 2025 Jan 23;487(3):701–11. doi: 10.1007/s00428-025-04028-2 (PMC12488736; doi:10.1007/s00428-025-04028-2)

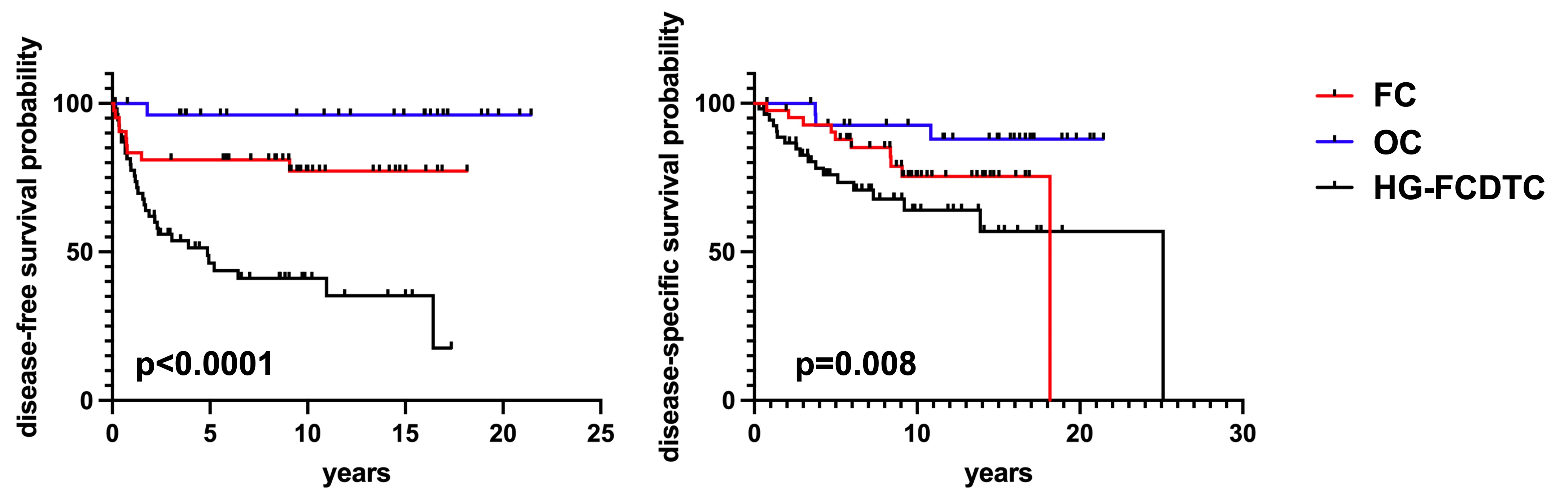

Supplement: Supplementary file 2 — Supplementary file2 (TIF 1087 KB) [file 428_2025_4028_MOESM2_ESM.tif]
